# Assessment of biosafety precautions in Khartoum state diagnostic laboratories, Sudan

**Published:** 2012-02-03

**Authors:** Adel Hussein Elduma

**Affiliations:** 1 National Public Health Laboratory, Ministry of Health, Khartoum, Sudan

**Keywords:** Biosafety, Diagnostic laboratories, assessment, Sudan

## Abstract

**Background:**

This study was conducted to evaluate the biosafety precautions that applied by diagnostic laboratories in Khartoum state, 2009.

**Methods:**

A total number of 190 laboratories were surveyed about their compliance with standard biosafety precautions. These laboratories included 51 (27%) laboratories from government, 75 (39%) from private sectors and 64 (34%) laboratories belong to organization providing health care services.

**Results:**

The study found that 32 (16.8%) of laboratories appointed biosafety officers. Only, ten (5.2%) participated in training about response to fire emergency, and 28 (14.7%) reported the laboratory accident occurred during work. 45 (23.7%) laboratories had a written standard operation procedures (SOPs), and 35 (18.4%) had written procedures for the lean-up of spills. Moreover, biosafety cabinet was found in 11 (5.8%) laboratories, autoclave in 28 (14.7%) and incinerator in only two (1.1%) laboratories. Sharp disposable containers were found in 84 (44.2%). Fire alarm system was found in 2 (1.1%) laboratories, fire extinguisher in 39 (20.5%) laboratories, and fire emergency exit found in 14 (7.4%) laboratories. Furthermore, 19 (10%) laboratories had a hepatitis B virus vaccination programme, 5 (6.2%) applied BCG vaccine, and 2 (1.1%0) vaccinated the staff against influenza.

**Conclusion:**

The study concluded that the standards biosafety precautions adopted by the diagnostic laboratories in Khartoum state was very low. Further, the laboratory personnel awareness towards biosafety principles implementation was very low too.

## Background

Biosafety is described as a safe method for managing infectious agents in laboratory environment where they are handled and maintained. Implementation of biosafety precautions decreases the exposure to the risk factors inside the laboratory. There are four main biosafety levels for laboratories designated as; level-1 basic, level-2 containment, level-3 and maximum containment is level-4 [1]. Diagnostic laboratories located in public health centers, clinics and hospital institutions and dealing with infectious materials are considered as a high risk area for staff working in it [2]. Many types of events can take place in laboratories and cause infection. These hazards include the following; infectious aerosols, spills, needles stick injuries, cuts from sharp objects, broken glass, chemical and radioactive materials, centrifuge accidents and fire. Individuals who work in these laboratories and handle infectious materials are at high risk to get infection [3]. In addition to that, laboratory staff exposed to chemical and radioactive materials, flammable gases, electrical accidents and fire hazards [4]. Laboratory acquired infections are a common problem all over the world and many cases have been reported [5]. In 1949, Sulkin and Pike published the first serious surveys of laboratory associated infections. Since 1980s, laboratories have applied fundamental guidelines in activities associated with blood borne pathogens [6]. In addition to that, Harding and Byers indicated that 45% to 51% of laboratories associated infections took place in clinical, diagnostics and research laboratories [7]. Standards precautions such as gloves wearing, hands washing, safety glasses and face shield is highly recommend in diagnostic laboratories. In a study conducted in Maryland State, United States of America, found that compliance with universal precautions in health care facilities was reported as low rate for certain types of personal protective equipment such as protective eye wear, face mask and protective clothing [8].

So, biosafety precautions in diagnostic laboratories become a crucial issue that should be followed. These precautions included the practices, safe equipment and facility, protection of laboratory staff and public environment from exposure to infectious substances. We conducted this study to evaluate biosafety precautions which adopted by diagnostic laboratories in Khartoum state.

The general objective of this objective of this study was to evaluate the biosafety precautions adopted in diagnostic laboratories in Khartoum State. The specific objectives of this study were: 1) to investigate laboratory biosafety standards practices and techniques in diagnostic laboratories; 2) to determine the awareness of laboratory staff towards potential hazards.

## Methods

**Study design**: Cross-sectional study.

**Study setting:** The study was conducted in Khartoum state diagnostic laboratories.

**Study period**: December 2008-December 2009.

**Study subject:** Diagnostic laboratories in Khartoum state represented as study subject which included laboratories belonged to government, private institutions and organization providing health services.

**Sample size**: Sample was calculated according to the sample size equation N = Z^2^pq/d^2^, n = number of study population participated in the study, Z = constant, p= previous data, q = 1-p, d = level of confidence

**Sampling technique:** Stratified simple random sampling technique was used in selecting laboratories.

**Data collection:** A designed questionnaire and checklist were used to collect data from laboratories. Many variables were involved in these two data collection tools. Variables were characterized in to; variables for safety precaution measures at workplace, variables for personal protection equipment, and variables for services provided inside laboratory. In addition to that, variables of essential biosafety equipment, risk determination, fire prevention and vaccination programme were also included in data collection tools.

Data entered and analyzed by statistical package SPSS (Statistical Package for Social Science)

When data collection phase was finished, visits were made to laboratory participation in this study. The aim of these visits was to check the accuracy of collected data. Any wrong information in the questionnaire of the checklist was corrected. Information collected from labs revised by supervision visits. The main objective of these visits was to check the accuracy of data gathered during the collection mission. Any wrong data corrected before entered into the data analysis programme.

## Results

A total number of 190 laboratories (labs) were surveyed about their compliance with standard biosafety precautions. These laboratories included 51 (27%) labs from government, 75 (39%) from private sectors and 64 (34%) labs belong to organization providing health care services. The study found that 32 (16.8%) of labs appointed biosafety officers, while 75 (39.5%) working in these laboratories indicated that they attended biosafety training previously. Government laboratories were more likely to train their staff when compared to other private and health organization laboratories, still this different was not significant (P= 0.145).

Only 20 (10.5%) labs inspected gas cylinders regularly 10 (5.2%) had previous training in response to fire emergency and 28 (14.7%) reported the occurrence of laboratory accident in the work. Furthermore, 45 (23.7%) labs had a written standard operation procedures (SOPs), and 35 (18.4%) had written procedures for the lean-up of spills (Table 1). Of the total number, 112 (58.9%) lab staff usually wear lab-coat in all labs procedures, 72 (37.9) some time do that and 6 (3.2%) never wear it during work time. Regarding eating and drinking inside the labs, only 6 (3.2%) of lab staff do that usually .117 (61.6%). Of the investigated labs stated that gloves worn in all laboratory procedures and 166 (87.4%) labs indicated that they practiced hand washing before and after each laboratory procedures (P=0.014).

**Table 1:** Finding of laboratory basic biosafety precautions assessment in 190 laboratories (Government, Private and Health Organization) in Khartoum state, Sudan

	Appointment of biosafety officer			SOPs			Training on Biosafety			Gas cylinder and valve regularly inspected			Hand wash in all laboratory procedures		
	
	Yes	No	p	Yes	No	p	Yes	No	p	Yes	No	p	Yes	No	p
Government	25	26	0.000	18	33	0.000	26	25	0.143	10	41	0.017	47	4	0.014
Private	5	70		4	71		26	49		8	67		59	16	
Organization	2	62		0	64		23	41		2	62		60		

	**Written report of spills and accidents**			**Personnel trained in fire emergency**			**Entry restriction to the laboratory**			**Reporting of laboratory accident**			**Using of automatic pipette**		
	
	**Yes**	**No**	**p**	**Yes**	**No**	**P**	**Yes**	**No**	**p**	**Yes**	**No**	**p**	**Yes**	**No**	**p**

Government	5	46	0.026	8	43	0.000	25	26	0.001	18	33	0.000	45	6	0.002
Private	3	72		2	73		34	41		3	72		70	5	
Organization	2	62		0	64		12	52		7	57		46	18	

Drinking water was available in 172 (90.5%) labs and toilet for both male and female were provided in 99 (52.1%).

**Figure 1:**
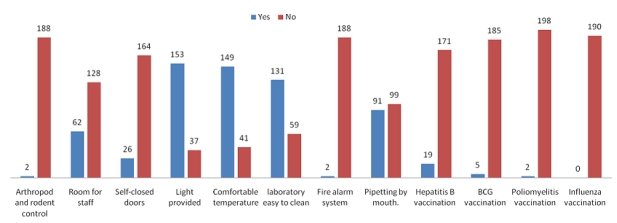
Biosafety precautions in work area and vaccination programme for staff working in Khartoum State diagnostic laboratories, Sudan

Government laboratories were more like to train their staff. Reporting of laboratory accident was very low, only 28 (14.7%) laboratories did that (p= 0.000). Also, entering to the laboratories was investigated and government labs were found more restricted than other laboratories. Moreover 147 (78.4%) lab staff indicated that working temperature was comfortable, and 131 (68.9%) of them said that ceiling and floors were easy to clean (Figure 1).

Only 24 (12.6%) laboratories had separated room for sampling and 57 (30%) had special room for patients. Essential biosafety equipment was also investigated and only 14 (7.35%) labs used self needle device in the work. In addition, biosafety cabinet was found in 11 (5.8%) labs, autoclave in 28 (14.7%), and incinerator in only 2 (1.1%) labs. Sharp disposable containers were found in 84 (44.2%) labs, but the difference between government, private and organization laboratories was not significant (P=0.149). Also, only 5 (2.6%) laboratories had waste container chemical materials and 4 (2.1) had radioactive waste containers.

Fire alarm system was found in 2 (1.1%) laboratories, fire extinguisher in 39 (20.5%) labs, and fire emergency exit found in 14 (7.4%) labs. Of the laboratories, 47(24.7%) indicated that they kept gas cylinder away from heat sources (Table 2).

**Table 2:** Basic biosafety precautions at work place and vaccination programmes for staff working in 190 laboratories (Government, Private and Health Organization) in Khartoum state, Sudan

	Safe needle device			Separated room for sampling			Staff room for eating and drinking			Automatic pipette availability			Autoclave device		
	
	Yes	No	p	Yes	No	p	Yes	No	p	Yes	No	p	Yes	No	P
Government	9	42	0.002	22	29	0.000	18	33	0.545	45	6	0.174	19	32	0.000
Private	5	70		10	65		21	54		56	19		7	68	
Organization	0	64		2	62		23	41		51	13		2	62	

	**Radioactive waste container**			**Fire emergency exit**			**Self-closed door**			**Sharp disposable container**			**Availability of extinguisher**		
	
	**Yes**	**No**	**p**	**Yes**	**No**	**Yes**	**Yes**	**No**	**p**	**Yes**	**No**	**p**	**Yes**	**No**	**p**

Government	4	47	0.004	12	39	0.000	15	36	0.001	27	24	0.149	24	27	0.000
Private	0	75		2	73		5	70		27	48		7	68	
Organization	0	64		0	64		5	58		30	34		1	63	

Furthermore, 19(10%) laboratories had hepatitis B virus vaccination programme, 5 (6.2%) applied BCG vaccine, and 2 (1.1%) vaccinated the staff against influenza.

## Discussion

This study was investigating the compliance of diagnostics laboratory to standard biosafety precautions. The study found that laboratory staff did not wear lab-coat and gloves in all laboratory procedures as the percentage was very low. Also, personnel working in these diagnostics laboratories did not receive the required training in biosafety. In addition, reporting system for laboratory accidents and clean-up of spills was not completely carried out. The percentage of these parameters was low when compared to a similar study in Turkey [9].

The low percentage of benches cleaning, hand wash found in this study may increase the risk of infected with pathogenic agents inside these laboratories. Further laboratory work area precautions were also investigated and only 13(6.8%) laboratories were using international biohazard symbols and signs, while 71 (37.4 %) of them had an entry restrictions to their site. No doubt, application of these precautions will enable laboratory personnel to reduce the risk of acquiring infection or export the infection to the surrounded environment [10].

In study conducted in laboratories across Canada to determine rates of compliance with recommended safety precautions against exposure to blood borne pathogens. The study found that laboratory personnel are highly exposure to body fluids and poor rate compliance with personal protective behaviors, this finding is similar to our study results [11]. Essential equipment such as biosafety cabinet, autoclave, incinerator and screw caped container and bottles are so important to safe work area. Although the number of Biosafety cabinets was very low 11 (5.8%) when compared with study conducted in Japan, where 70% of surveyed laboratories had biosafety cabinet. Although the biosafety cabinet number was low, it found only in the government and private laboratories, no one was reported in laboratories belong to organization provide health services. All these biosafety cabinets were either level -1 or level -2, no laboratory had biosafety cabinet level-3 [12]. In addition, the percentage of incinerator in our study was low when compared to the study conducted in Pakistan. There were only two incinerators, the first one of them belonging to government laboratory and the second is a private laboratory. The number of waste and sharp disposable containers and was not significant when compared to the ideal number that should be provided for laboratories [13]. Laboratory staff is at high risk of acquiring infectious diseases including vaccine preventable diseases. Besides, implementation of biosafety precautions, it is essential to provide immunity from vaccine preventable diseases such as hepatitis B virus, tuberculosis, influenza, and poliomyelitis. Provision of vaccines for these diseases was investigated, but the percentage of vaccination programme was very low for example only 19 (10) of these labs had a policy for hepatitis B virus vaccination [14].

## Conclusion

This study indicated that the standards biosafety precautions adopted by the diagnostics laboratories in Khartoum state was very low. In addition to that, awareness of laboratory personnel towards biosafety principles implementation was very low too.

## References

[1] (1989). Safe handling of infectious agents. Biosafety in the Laboratory, Prudent Practices for the Handling and Disposal of Infectious Material.

[2] (2003). Laboratory biosafety manual.

[3] Mandell GL, Bennett JE, Dolin R (2000). Principles and practice of infectious diseases.

[4] (1999). Biosafety in microbiological and biomedical laboratories.

[5] Sewell DL (1995). Laboratory-Associated Infections and Biosafety. Clin Microbiol Rev.

[6] Sulkin SE, Pike RM (1951). Survey of Laboratory-Acquired Infections. Am J Public Health Nations Health.

[7] Harding AL, Byers KB, Fleming DO, Hunt DL (2000). Epidemiology of laboratory-associated infections. Biological safety: principles and practices.

[8] Gershon RR, Karkashian CD, Vlahov D, Kummer L, Kasting C, Green-McKenzie J, Escamilla-Cejudo JA, Kending N, Swetz A, Martin L (1999). Compliance with universal precautions in correctional health care facilities. Journal of occupational and environmental Medicine..

[9] Aksoy U, Ozdemir MH, Usluca S, Toprak Ergönen A (2008). Biosafety profile of laboratory workers at three education hospitals in Izmir, Turkey. Mikrobiyol Bul.

[10] Richmond JY, Nesby-O'Dell SL (2002). Laboratory security and emergency response guidance for laboratories working with select agents. Centers for Disease Control and Prevention. MMWR Recomm Rep.

[11] Main CL, Carusone SC, Davis K, Loeb M (2008). Compliance with personal precautions against exposure to blood borne pathogens among laboratory workers: a Canadian survey. Infection Control and Hospital Epidemiology..

[12] Goto M, Yamashita T, Misawa S, Komori T, Okuzumi K, Takahashi T (2007). Current biosafety in clinical laboratories in Japan: report of questionnaires' data obtained from clinical laboratory personnel in Japan. Kansenshogaku Zasshi..

[13] Abdul Mujeeb S, Adil MM, Altaf A, Shah SA, Luby S (2003). Infection control practices in clinical laboratories in Pakistan. Infection control and hospital epidemiology..

[14] Rusnak JM, Kortepeter MG, Hawley RJ, Anderson AO, Boudreau E, Eitzen E (2004). Risk of occupationally acquired illnesses from biological threat agents in unvaccinated laboratory workers. Biosecur Bioterror..

